# Comparing Morphometric and Mitochondrial DNA Data from Honeybees and Honey Samples for Identifying *Apis mellifera ligustica* Subspecies at the Colony Level

**DOI:** 10.3390/ani15121743

**Published:** 2025-06-12

**Authors:** Valeria Taurisano, Anisa Ribani, Valerio Joe Utzeri, Dalal Sami, Kate Elise Nelson Johnson, Giovanni Formato, Marcella Milito, Giuseppina Schiavo, Samuele Bovo, Francesca Bertolini, Luca Fontanesi

**Affiliations:** 1 Animal and Food Genomics Group, Division of Animal Sciences, Department of Agricultural and Food Sciences, University of Bologna, Viale Giuseppe Fanin 46, 40127 Bologna, Italy; valeria.taurisano2@unibo.it (V.T.); anisa.ribani2@unibo.it (A.R.); va.joe6@gmail.com (V.J.U.); dalal.sami@unibo.it (D.S.); kate.johnson@columbia.edu (K.E.N.J.); giuseppina.schiavo2@unibo.it (G.S.); samuele.bovo@unibo.it (S.B.); francesca.bertolini3@unibo.it (F.B.); 2 Istituto Zooprofilattico Sperimentale del Lazio e della Toscana “M. Aleandri”, Via Appia Nuova 1411, 00178 Roma, Italy; giovanni.formato@izslt.it (G.F.); marcella.milito@izslt.it (M.M.)

**Keywords:** apiculture, beekeeping, conservation genetics, genetic integrity, morphometry, mtDNA, population genetics

## Abstract

The ability to differentiate between different *Apis mellifera* subspecies can have significant implications for their conservation. In this study, we explored the possibility of obtaining multi-level information that could be useful in assigning a colony to the *A. m. ligustica* subspecies. This was accomplished by combining (1) standard morphometric characteristics of the honeybees with mitochondrial DNA (mtDNA) sequence data obtained from both the (2) honeybees and (3) honey present in a honeycomb sampled from the same colony. Eighty colonies were sampled in the Emilia-Romagna region (Northern Italy). From these colonies, a total of 413 honeybees were analyzed to obtain sequence information of the tRNALeu-COII mtDNA region. When we combined the results obtained from all three methods, 63.75% of the colonies were found to be compatible with being assigned to *A. m. ligustica*. For 36.25% of the colonies, morphometric analysis indicated a hybrid origin and mtDNA from honeybees or honey samples indicated the presence of mtDNA haplotypes other than those characteristic for this subspecies. Cohen’s Kappa statistic indicated poor agreement in the classification of the colonies between morphometric analysis and the two DNA-based methods, which, in turn, were in substantial agreement with each other. Although not completely effective, mtDNA analysis of honey samples could serve as an initial and practical compromise to begin initiatives aimed at preserving the genetic integrity of *A. m. ligustica* in the Emilia-Romagna region, as well as in other regions where it is necessary to preserve the genetic integrity of autochthonous honeybee populations.

## Simple Summary

Assigning a honeybee colony to the corresponding *Apis mellifera* subspecies is crucial for implementing conservation programs that preserve the genetic integrity of local populations, even though it can be technically and economically challenging when large-scale monitoring approaches are needed. Morphometric analysis of honeybees, considered the gold standard for this purpose, is a demanding method in terms of sample preparation. Therefore, we compared the results obtained from this method with findings from analyzing mitochondrial DNA (mtDNA) from honeybees and honey sampled from the same colonies used for morphometric analysis. To comparatively assess these three methods, we took advantage of a field study derived from the need to apply a regional law issued by the Emilia-Romagna region (Northern Italy) protecting the *A. m. ligustica* subspecies. For approximately two-thirds of the eighty colonies that we analyzed, all three methods agreed on classifying the colonies as either belonging to the *A. m. ligustica* subspecies or not. Although not completely effective in terms of informativeness, the analysis of mtDNA from honey samples could serve as an initial cost-effective assay useful to begin initiatives aimed at assessing the genetic integrity of *A. mellifera* subspecies.

## 1. Introduction

A total of approximately 30 subspecies, primarily distinguished based on different morphometric characters, have been described in *Apis mellifera* (Linnaeus, 1758), originally distributed in their corresponding native regions that include Europe, Africa, and Western Asia [1,2,3]. These subspecies are believed to be well adapted to their natural environments. The conservation of their genetic integrity is, therefore, considered very important for the long-term sustainability of the beekeeping sector and to maintain the associated ecosystem services, such as pollination [4,5,6,7]. Several initiatives have been proposed in various European countries to preserve honeybee genetic resources. This comes after evidence has shown that many honeybee subspecies are losing their genetic distinctiveness [5,8,9].

The ability to differentiate between different honeybee subspecies can have significant implications for their conservation [10,11,12]. Standard morphometry, which involves measuring various body parts of worker bees, such as wing characters, color, and pilosity, is the traditional method used to distinguish between subspecies [1,2,13,14,15]. These morphometric measurements are then typically elaborated with multivariate analysis to assign bees collected from a colony with a certain level of confidence to a reference subspecies [2]. DNA analysis of honeybees has also been suggested as a complementary or alternative method for identifying subspecies [2,3,16,17,18]. Certain mitochondrial DNA (mtDNA) lineages and, within lineages, a few mtDNA haplotypes have consistently been associated with morphometrically assigned and geographically distributed subspecies, with some overlap between the DNA-derived information and morphometric information [1,2,3,19,20]. This indicates that the honeybee mtDNA lineage may be useful in subspecies identification or exclusions.

Several main mtDNA lineages have been reported, along with their corresponding subspecies groups, some of which are considered endemic in Italy [1,21,22,23,24]. Among them, lineage A, the African lineage, is mainly found in subspecies originating from Africa and that colonized several Mediterranean islands (including Sicily, with *A. m. siciliana*) and other South European regions (such as the Iberian Peninsula, with *A. m. iberiensis*). The mtDNA lineage C is present in subspecies mainly found in Southeastern European regions [3,24], for example, *A. m. ligustica*, which is considered the main endemic Italian subspecies, and *A. m. carnica*, found in the Western Balkan regions and also present in the East Alpine arch areas and nearby Italian regions. The mtDNA M lineage is found in *A. m. iberiensis*, together with the mtDNA A lineage, as the Iberian Peninsula is a contact point of African and Western European evolutionary lineages [25,26]. The mtDNA lineage M is one of the primary genetic characteristics of *A. m. mellifera* subspecies, mainly spread in Western and Northern Eurasia, from the British Isles through most of continental Europe, to the Ural Mountains and some areas in Central Asia. *Apis mellifera mellifera* is also considered to be endemic in the north–west borders of Italy, including parts of the Liguria region and nearby Piedmont Alpine areas [1,3,4,5,12,21,27]. *Apis mellifera ligustica* (Spinola, 1806), in addition to the C1 haplotype, has been reported to carry mtDNA haplotypes of the M3/M7 group, as a result of its evolutionary history where M branches found refuge in the Italian Peninsula during the Quaternary ice period [24].

In Italy, the Emilia-Romagna region, located in the northern part of the Italian Peninsula and bordered by the Po River in the north and by the Apennine Mountains in the south, has been the first regional authority to regulate the conservation of the *A. m. ligustica* subspecies [28]. Article No. 7 of the Regional Law No. 2, dated 4 March 2019 (Regione Emilia-Romagna, 2019), prohibits the breeding and introduction of subspecies other than *A. m. ligustica* in this region [28]. This regional legislative act is significant because the region is home to one of the largest concentrations of queen breeding activities for this native honeybee subspecies in Italy. Therefore, it is becoming increasingly important to be able to identify this subspecies accurately using various sources of information and biological specimens. This is necessary in order to monitor the application of this regional law in a cost-effective and technically efficient manner.

We have recently demonstrated the feasibility of extracting DNA from honey and analyzing it to determine the mtDNA lineage of the honeybees that produced it [29,30,31]. This method can be used on honey found in honeycombs, establishing a connection with the colony where the honey is produced. If applied to honey that has been separated from the honeycombs, this link is lost, even though it could maintain a link at the apiary or beekeeper levels with appropriate labels on honey jars [30]. It is also widely recognized that beekeeping practices often alter the composition and origin of the honeybee populations within a colony, such as through the sharing of honeycombs and merging nuclei, complicating the application of monitoring assays at the hive level.

In this study, we explored the possibility of obtaining multi-level information that could be useful in identifying the subspecies of honeybees within a colony. This was accomplished by combining morphometric characteristics of the honeybees with mtDNA sequence data obtained from both the honeybees and honey present in a honeycomb, sampled from the same hive. We then assessed the consistency of these different layers of information through a field trial, which offered valuable insights into practical procedures for monitoring the subspecies of honeybees within a colony.

## 2. Materials and Methods

### 2.1. Honeybees and Honey Samples

This study was conducted by analyzing 80 samples of honeybees, each collected from a different colony. Each sample was constituted by approximately 40 young workers, captured with a 50 mL tube from the brood frame. The tubes contained about 35 mL of pure ethanol and were then stored at +4 °C until subsequent analyses. Honeybees were used for morphometric characterization and for DNA analyses, as described below. From the same 80 colonies, honeycombs (approximately 10.0 × 10.0 cm) with stored honey were also sampled at the same time as worker bee collection. Honeycombs were inserted into freezer food storage plastic bags and then stored at +4 °C until subsequent analyses. Honey samples were used for DNA analyses, as detailed below.

**Figure 1:**
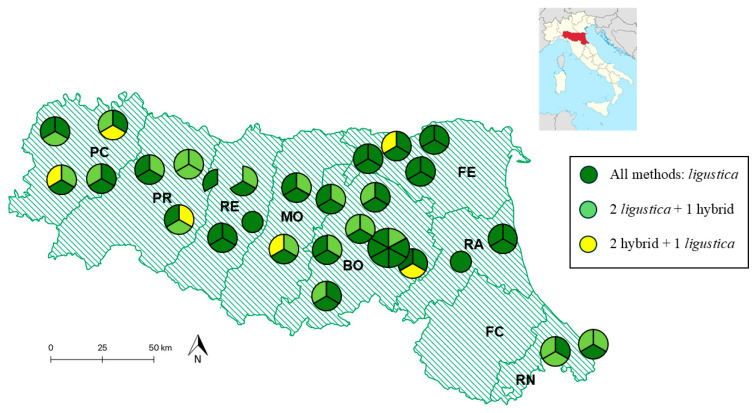
Geographical distribution of the apiaries of the Emilia-Romagna region where honeybees and honeycombs were sampled. The apiaries are represented by circles, including sections of various colors: each section indicates the presence of colonies that were either “compatible” or “non-compatible” (“hybrid”) with the assignment to the *A. m. ligustica* subspecies using the three methods described in this study. Dark green circles or sections represent colonies that were “compatible” with the assignment to *A. m. ligustica* using all three methods. Pale green circles or sections indicate colonies that were “compatible” with the assignment using two methods, but “non-compatible” or “hybrid” according to the third method. Yellow sections represent colonies that were “compatible” with one method, but not with the other two. The circles are divided into various sections based on the number of colonies sampled in the same apiary. In one case, a beekeeper provided colonies from two apiaries, which are shown as two sections of the same circle. The administrative borders of the provinces (indicated with acronyms) in the Emilia-Romagna region are also shown. The acronyms for the provinces are as follows: Piacenza (PC), Parma (PR), Reggio Emilia (RE), Modena (MO), Bologna (BO), Ferrara (FE), Ravenna (RA), Forlì-Cesena (FC), and Rimini (RN). The position of the region in Italy is indicated in the subset.

All samples were collected in 2020 and derived from 28 different apiaries, owned by 27 different beekeepers (1 beekeeper provided 1 colony from 1 apiary and 2 other colonies from another apiary). Sampling was considered to be random and not biased, as it was only based on the willingness of beekeepers to collaborate to this research. Most beekeepers (n = 24) provided 3 samples each, 2 beekeepers provided 1 sample each, and 1 beekeeper provided 6 samples. Sampled colonies were randomly selected among the colonies of the same apiary that accounted for 20 to 65 colonies each. All apiaries were in the Emilia-Romagna region. Figure 1 shows the geographic positions of the 28 apiaries. Detailed information on all 80 colonies is reported in Table S1.

### 2.2. Morphometric Analysis of the Honeybees

From the collected young worker bees, a sample of 20 individuals was then used for morphometric analysis. Upon arrival in the laboratory, the right forewings of each of the 20 honeybees were removed and placed flat on a photographic slide to obtain 2 columns of 10 wings each. Subsequently, the heads and thoraxes were removed and the second and third tergites were analyzed by comparing the width of the yellow band with the “Goetze Scale” [15]. The process began by examining the slide under a stereomicroscope to measure the width and length of each wing. The slide was then scanned into a computer, and each wing was digitally isolated using Adobe Photoshop CS6 (Adobe, San Jose, CA, USA). Next, the DataBees software [32] was used to measure 19 specific landmark points (which are specific junction points between the wing veins; Figure S1) on each wing [2,3,32]. These points were manually acquired and stored in the DataBees software [32]. The software incorporates the DAWINO (Discriminant Analysis with Numerical Output) method [3,33], which analyzes parameters of the forewing. The landmark points were used as references to calculate additional measurements that aided in identifying the bee subspecies, including a total of 30 parameters based on angles, ratios, linear measurements, indices, and areas [2,3]. The abdominal portions of the same worker bees were also used to obtain information on the color of the tergites, following Goetze’s classification [15], in accordance with the Italian procedure previously described [2].

### 2.3. DNA Extraction

DNA was extracted from individual honeybees from a leg using the Wizard Genomic DNA Purification Kit (Promega, Promega Corporation, Madison, WI, USA), following the manufacturer’s protocol for animal tissues.

DNA extraction from honey samples was carried out following the protocol described by Utzeri et al. [29]. Honey was first separated from the honeycombs by gravity after elimination of the beeswax. Then, each sample was first pre-treated to eliminate the sugar components with a series of dilutions with water, followed by centrifugation steps. The obtained pellets were resuspended in 0.5 mL of sterile water and added with an extraction buffer composed of 1 mL of CTAB buffer (2% (*w*/*v*) cetyltrimethylammonium bromide: 1.4 M NaCl, 100 mM Tris-HCl, 20 mM EDTA, pH 8.0), 5 µL of RNase A solution (10 mg/mL), and 30 µL of proteinase K solution (20 mg/mL). The tube containing the sample and the extraction buffer was incubated at 65 °C for 90 min and mixed gently. After this step, the tube was centrifuged at 16,000× *g* for 10 min at room temperature. A total of 700 µL of the obtained supernatant was transferred in a new tube and added with 500 µL of chloroform/isoamyl alcohol (24:1) solution. All tubes were vortexed for 30 s and then centrifuged at 16,000× *g* for 15 min at room temperature. The supernatant was transferred into a new 1.5 mL tube for DNA isolation and purification steps with isopropanol and ethanol 70%. Honey DNA was finally resuspended with 30 µL of sterile water and stored at −20 °C until PCR analyses.

DNA extracted from both individual honeybees and honey samples was evaluated by electrophoresis on TBE 1× 1% agarose gels stained with 1× GelRed Nucleic Acid Gel Stain (Biotium Inc., Haward, CA, USA) and quantified using a Nanophotometer P-330 instrument (Implen GmbH, München, Germany).

### 2.4. PCR Analyses and Sequencing

#### 2.4.1. Analyses on Honeybee DNA

The primer pair designed by Garnery et al. [16] (E2, forward: 5′-GGCAGAATAAGTGCATTG-3′; H2, reverse: 5′-CAATATCATTGATGACC-3′) was used to amplify DNA extracted from bee workers. These primers amplified a standard fragment of the mtDNA that is routinely used to discriminate between different *A. mellifera* mtDNA haplotypes. The amplified region included a portion of the intergenic non-coding region of the mtDNA located between COI and COII (or *cox1* and *cox2* genes) that contains the tRNA-Leu sequence (tRNALeu-COII). PCR was obtained in an Applied Biosystem SimpliAmp Thermal Cycler (Thermo Fisher Scientific Inc., Waltham, MA, USA), in a reaction volume of 14 µL. The amplification mix was constituted by 2× Kapa Hifi HotStart ReadyMix PCR kit (Kapa Biosystems, Boston, MA, USA), 10 pmol of each primer, and 20–50 ng of template DNA. The PCR amplification steps started with a denaturation step at 95 °C for 5 min, followed by 35 cycles of alternate temperatures (30 s at 95 °C, 30 s at 54 °C, and 30 s at 72 °C) and by a final extension step at 72 °C for 5 min. The amplified DNA fragments were then checked in 2.5% agarose gel TBE 1× buffer electrophoresis after staining with 1× GelRed Nucleic Acid Gel Station (Biotium Inc.).

PCR amplicons were purified with a standard isopropanol precipitation–purification protocol and subsequently used for Sanger sequencing. Sequencing reactions were obtained with the chain termination protocol of the BrightDye terminator cycle sequencing kit (NIMAGEN, Nijmegen, The Netherlands) and loaded on an ABI3100 Avant Genetic Analyzer Sequencer (Applied Biosystems, Foster City, CA, USA).

For each sampled colony, sequencing information was obtained from 1 (for 42 colonies) or more than 1 worker bee (for the remaining colonies, from 7 to 17 workers bees; Table S1), to have an estimation of the sampling bias derived from analyzing a limited number of randomly selected bees from a colony. The 38 colonies from which more than 1 honeybee was analyzed were divided in the same proportion with the morphometric classification of the whole colonies: approximately 21% (8 out of 38) were classified as hybrids, while the others were classified as belonging to *A. m. ligustica*.

#### 2.4.2. Analyses on Honey DNA

Due to the high degradation level of the DNA extracted from honey, only short amplicons can be obtained from this matrix [29]. Here, the primer pair (forward 5′-GGCAGAATAAGTGCATTG-3′and reverse 5′-TTAATATGAATTAAGTGGGRAAW-3′) and PCR conditions previously reported by Utzeri et al. [29] were used to produce amplicons that identify which of the three main *A. mellifera* mtDNA lineages (A, C, and M) was carried out by the honeybees that produced the analyzed honey samples. These three mtDNA lineages can be differentiated based on the amplified fragment size (152 bp for the A lineage, 138 bp for the M lineage, and 85 bp for the C lineage [29,30]) after 4% agarose gel electrophoresis in TBE 1× buffer and staining of the DNA bands with 1× GelRed Nucleic Acid Gel Stain (Biotium Inc.). To discriminate C1 and C2 mtDNA lineages from honey DNA, another primer pair (forward: 5′-GGCAGAATAAGTGCATTG-3′; reverse: 5′-ATCTTTAAGATTGAATTTAAAATTC-3′ [31]) was used with the amplification conditions already reported [31]. The obtained amplicons were Sanger sequenced, as described above.

### 2.5. Data Analyses

#### 2.5.1. Morphometric Data Analyses

From the 20 wings analyzed, as described above, the mean and standard deviation were calculated for each parameter. These data were compared with a reference database that includes subspecies from Italy and neighboring countries for a discriminant analysis. The color of the tergites was considered separately. According to the Disciplinare dell’Albo Nazionale degli Allevatori di Api Italiane—Regulation of the National Register of Italian Honeybee Breeders [34]—*A. m. ligustica* exhibits pigmentation of the third tergite with a Goetze class [15] mean value of 7.65 and a standard deviation of 0.73. Morphometric values (mean and standard deviation) used for the identification of the *A. m. ligustica* subspecies are reported in Table S2. The reference database used by the DataBees software [32] to determine whether the examined sample conforms to a specific subspecies is based on 168 reference samples of *A. mellifera*, divided into 6 subspecies: *A. m. carnica* (n = 38), *A. m. ligustica* (n = 41), *A. m. mellifera* (n = 28), *A. m. intermissa* (n = 19), *A. m. caucasica* (n = 20), and *A. m. siciliana* (n = 22). After comparing the parameters of the samples under examination with the reference values from DataBees [32], the “posterior probability” was obtained, through a multivariate analysis using the Statistica software (ver. 7 Stat Soft Italia S.R.L., Tulsa, OK, USA). A scatterplot of the discriminant functions was then obtained, showing the areas where the different populations of *A. mellifera* fell. If the sample in question fell within the confidence ellipse of one of the subspecies of *A. mellifera*, with a 95% coefficient, the sample was considered consistent with that subspecies, also taking into account the coloration of the tergites, which must match the expected coloration according to the Goetze scale for that subspecies [15].

#### 2.5.2. Sequence Data Analyses

Sequence data quality was monitored by visually inspecting the obtained electropherograms using MEGA11 software [35]. Sequence data obtained from honeybees were aligned using the same software to build a multiple alignment based on reference sequences of *A. mellifera* mtDNA retrieved from the GenBank database (http://www.ncbi.nlm.nih.gov/nucleotide/, accessed on 20 January 2025). This alignment was used to identify the units of the polymorphic mtDNA region spanning the tRNAleu and COII genes that make it possible to classify the different mtDNA haplotypes [36]. The same sequences were analyzed using the BLASTN tool (http://www.ncbi.nlm.nih.gov/BLAST/, accessed on 20 January 2025), which compared the obtained sequences with those already available in GenBank for their assignment validation to different *A. mellifera* mtDNA lineages and haplotypes [36]. To further validate the classification of the mtDNA haplotypes, Geneious Prime 2022.0.1 software (https://www.geneious.com) was used to infer in silico *Dra*I profiles, which match sequence data with the classical *Dra*I mtDNA test, largely used before sequence information substituted this PCR-RFLP assay, previously regarded as the standard method for mtDNA haplotype definition [36,37]. Sequence data used to discriminate between C1 and C2 haplotypes (distinguished by the presence/absence of one cytosine) from honey amplified DNA [31] were aligned and analyzed with MEGA11 software [35].

#### 2.5.3. Comparison of Results from Different Methods

The classification of colonies was harmonized by considering two categories for all approaches by aligning the results obtained with the morphometric analyses: (1) “compatible” with *A. m. ligustica* and (2) “non-compatible” with *A. m. ligustica* or “hybrid” between *A. m. ligustica* and other subspecies. For the two DNA approaches, if only the C1 haplotype or the M3/M7 haplotype was identified, the colony was assigned to be “compatible” with *A. m. ligustica* [24], while in all other cases the colony was considered not to be compatible with *A. m. ligustica* and was assigned as belonging to “non-compatible” with *A. m. ligustica* or “hybrid”, to align the classification with that of the morphometric analysis. This method was applied both for the tRNALeu-COII mtDNA sequences obtained from honeybees and to DNA information obtained from honey DNA.

Concordant and discordant matrices across the three methods were also constructed. Then, pairwise contingency tables were used to evaluate the classification outputs of the 3 different approaches, considering all 80 colonies (Dataset 1) or only colonies from which only 1 honeybee was analyzed to obtain the tRNALeu-COII mtDNA sequence (n = 42; Sub-dataset 2) or only colonies from which more than 1 honeybee was analyzed to obtain the same sequence information (n = 38; Sub-dataset 3). Cohen’s Kappa statistic was used to measure the agreement between pairs of methods (the two raters) in assigning colonies to the “compatible” and “non-compatible” classes. The strength of agreement between two raters was assessed following Landis and Koch [38]. For example, negative values close to zero indicate poor agreement between the considered pairs of methods, while values between 0.61 and 0.80 indicate substantial agreement. The R package VennDiagram v. 1.7.3 [39] was used to prepare Venn diagrams merging concordant and discordant colony classifications derived from the three methods.

Bayesian inference was used to calculate the posterior probability P(A|B) for all three datasets (Dataset 1 and Sub-datasets 2 and 3), defined here as the probability that a colony classified as “compatible” for *A. m. ligustica* using one approach is also “compatible” when using a second method P(B), given the prior probability of a colony being classified as “compatible” for *A. m. ligustica* with the first method P(A), and the likelihood of colonies being classified as “compatible” with the two methods P(B|A). This calculation follows the Bayes rule: P(A|B) = P(B|A) × P(A)/P(B).

## 3. Results

Results were obtained from three different levels, illustrated separately and subsequently matched and compared. These levels consisted of (i) morphometric data of honeybees sampled from 80 colonies, (ii) DNA sequence information regarding the tRNALeu-COII mtDNA region directly produced from honeybees sampled from the same colonies, and (iii) DNA information retrieved from honey samples, collected from the same colonies, regarding mtDNA fragments of the honeybees.

### 3.1. Overview of Morphometric Profiles of the Analyzed Honeybee Samples

**Figure 2:**
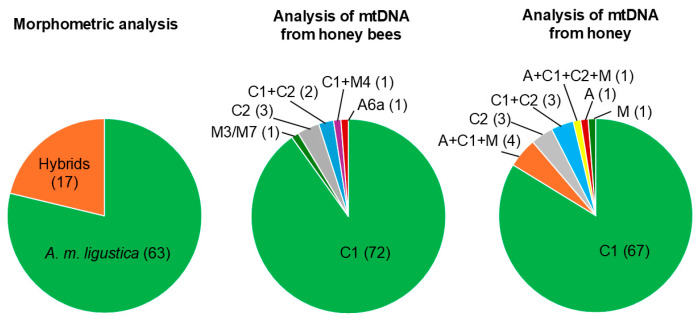
Proportion of colonies assigned to *A. m. ligustica* using the morphometric analysis and carrying different mtDNA haplotypes using the two DNA-based methods when considering Dataset 1. The number of colonies for each assignment and mtDNA haplotype are reported in parentheses.

Morphometric analyses classified 63 out of 80 colonies as “compatible” with *A. m. ligustica* (78.75%), while the remaining 17 colonies (21.25%) were classified as “non-compatible” with *A. m. ligustica* or “hybrids” between *A. m. ligustica* and other subspecies (Table 1; Figure 2).

Among the 27 beekeepers who provided samples, 12 (44.4%, including 2 beekeepers who provided just 1 sample each) had all colonies classified as “compatible” with *A. m. ligustica*. Another 12 beekeepers (44.4%) had two-thirds of the samples classified as “compatible” with *A. m. ligustica*. The remaining three beekeepers had only one sample classified as “non-compatible”. The apiaries where at least one sample was classified as “non-compatible” or “hybrid” were located in six out of eight administrative provinces of the Emilia-Romagna region. Among the provinces where colonies were sampled, colonies in the province of Ferrara (FE) and Ravenna (RA) were only classified to be “compatible” with *A. m. ligustica* (Figure 1).

### 3.2. Description of mtDNA Haplotypes of the Analyzed Honeybees

For practical applications in routine monitoring approaches, the same samples of honeybees collected for morphometric analysis were used for mtDNA analyses. DNA from a total of 413 honeybees was successfully amplified using the primer pair E2/H2 and then sequenced to obtain information on the tRNALeu-COII mtDNA region. The number of honeybee analyses for each colony ranged from 1 (n = 42 colonies) to more than 1 (n = 38 colonies). A total of five different mtDNA haplotypes were identified in the analyzed worker bees (Figure 2). The most frequent mtDNA haplotype was C1, identified in a total of 353 honeybees (85.5%). This mitotype is considered to be a characteristic of the *A. m. ligustica* subspecies [24,36,40]. The C2 mtDNA haplotype, considered to be specific to *A. m. carnica* [31,36,40], was identified in 31 analyzed worker bees (7.5%). Additionally, there were 10 sequences from haplotype A6a, 10 sequences from haplotype M3, and 9 sequences from haplotype M4. When considering Dataset 1 and grouping these sequences for each colony, we observed that 73 out of 80 colonies had mtDNA sequences that were “compatible” with being assigned to *A. m. ligustica*. However, seven colonies had honeybees with alien mtDNA sequences that were not considered original, either in all analyzed bees or at least one (Table 1; Figure 2).

Then, we dissected the whole dataset (Dataset 1) into two sub-datasets (Sub-datasets 2 and 3). Almost all sequences obtained from colonies where only one worker bee was analyzed (Sub-dataset 2) were from the C1 mtDNA haplotype (41 out of 42), and only in 1 case was the mtDNA haplotype C2. In 35 out of the remaining 38 colonies, where more than 1 worker bee was analyzed (ranging from 7 to 17 worker bees; Sub-dataset 3), all sequences derived from the same colony belonged to just 1 type of mtDNA haplotype: in 31 colonies, C1 was the only mitotype; in two colonies, C2 was the only mitotype; in one colony, A6a was the only one mitotype; in another colony, M3 was the only one mitotype. In 3 colonies where 10 worker bees were analyzed, we identified 1 prevalent mitotype (9 out of 10 sequences) with another minor haplotype. In one case, the prevalent mitotype was C1 and the minor was C2, in another colony it was the opposite, and in an additional colony the prevalent mitotype was M4 and the minor haplotype was C1. There was a significant difference (*p* < 0.05; two-tailed Fisher exact test) between the results obtained with Sub-datasets 2 and 3: when more honeybees were analyzed, it increased the chance of identifying an mtDNA haplotype that was not compatible with the assignment of the colony to *A. m. ligustica* (Table 1). Particularly in this sub-dataset, three colonies that had at least one honeybee with the C1 mtDNA haplotype (“compatible”) also had other worker bees with “non-compatible” mtDNA haplotypes.

Nineteen out of twenty-seven beekeepers consistently reported colonies with only the C1 mtDNA haplotype. The remaining beekeepers provided colonies in which we identified honeybees carrying different haplotypes, in addition to the C1 haplotype. However, the C1 haplotype was consistently detected as either a minor or major haplotype. These colonies were sampled from apiaries located in only five administrative provinces of the Emilia-Romagna region: Bologna (BO), Ferrara (FE), Modena (MO), Parma (PR), and Piacenza (PC; Figure 1).

### 3.3. Description of A. mellifera mtDNA Lineages Retrieved from Honey Samples

The analysis of the DNA extracted from honey samples with primers that discriminate the three main mtDNA lineages (A, C, and M) [29] indicated the prevalence of the C lineage, which was the only one detected in 73 out of 80 samples (91.2%). One honey sample contained only mtDNA of the A lineage and one honey sample contained only mtDNA of the M lineage. According to the definition of *A. m. ligustica* [24,36], the honeycomb containing only the A lineage was not compatible with being assigned to the *A. m. ligustica* subspecies. However, honeycomb containing only the M lineage was “compatible” with being assigned to the *A. m. ligustica* subspecies (with this method, we could not distinguish if the M lineage was M3/M7 or not). The remaining five honey samples reported all three bands in the gel electrophoresis analysis of the amplified fragments, indicating the co-presence of the A, C, and M lineages [29]. Then, all honey samples that reported the presence of the C lineage were also analyzed to distinguish between the C1 and C2 haplotypes [31]. A total of 71 honey samples contained only the C1 haplotype, 4 contained both C1 and C2 haplotypes, and 3 contained only the C2 haplotype. When combining these 2 analyses [31], a total of 68 (0.85) honey samples could be considered as being “compatible” with assignment to the *A. m. ligustica* subspecies (67 had only the C1 haplotype and 1 had only the M) and 12 had mtDNA haplotypes or multiple mtDNA haplotypes “non-compatible” for the assignment to *A. m. ligustica* (Table 1; Figure 2).

Nineteen beekeepers had all honey samples that contained only the C1 haplotype, three beekeepers had samples that contained both C1 and C2 haplotypes, and two beekeepers had one colony with only the C2 haplotype. The presence of three haplotypes (A, C1, and M) or four haplotypes (A, C1, C2, and M) was detected in honeycombs from colonies belonging to three beekeepers and one beekeeper (the same that also had colonies with honeycombs with three haplotypes), respectively. The apiaries where honey samples collected reported the presence of another mtDNA haplotype in addition to the C1 haplotype were located in five administrative provinces of the Emilia-Romagna region: Bologna (BO), Ferrara (FE), Modena (MO), Parma (PR), and Piacenza (PC; Figure 1).

### 3.4. Comparing Results Obtained from the Three Methods

When considering all analyzed colonies, the results on the number of “compatible” and “non-compatible” colonies were not statistically different (*p* > 0.05) in all pairwise comparisons between the three methods (Table 2). In the comparison between morphometric analysis and mtDNA analysis of the honeybees from the same colonies, there was a suggestive significant difference (*p* = 0.053) in declaring “compatible” and “non-compatible” *A. m. ligustica* colonies. The number of “non-compatible” colonies was higher using the morphometric method. This difference was further amplified in Sub-dataset 2 (*p* = 0.01), where only one honeybee was analyzed to determine the mtDNA haplotype (Table 2). Within this subset of colonies, significant differences were also observed between the morphometric method and mtDNA analysis of the honey samples (Table 2). When multiple honeybees were analyzed, there were no significant differences between the three methods (Table 2). Therefore, it is evident that the difference observed in Dataset 1 was largely attributed to a bias stemming from the limited informativeness when only one honeybee per colony was analyzed.

When we combined the results obtained from all three methods, which may provide complementary information, a total of 29 out of 80 colonies (36.25%) were found to be incompatible with being assigned to *A. m. ligustica*. These colonies exhibited “non-compatible” features for one or two methods. These colonies belonged to 20 out of 27 beekeepers (74%) involved in the study. The percentage of “non-compatible” colonies increased to 42.11% when we considered only the 38 colonies for which multiple honeybees were analyzed. Pairwise matches based on Dataset 1 revealed 71.25%, 65.00%, and 82.50% “compatible” colonies between the morphometric analysis and the tRNALeu-COII mtDNA region analysis of the honeybees, the morphometric analysis and the mtDNA analysis of the honey, and the two mtDNA analyses, respectively.

**Figure 3:**
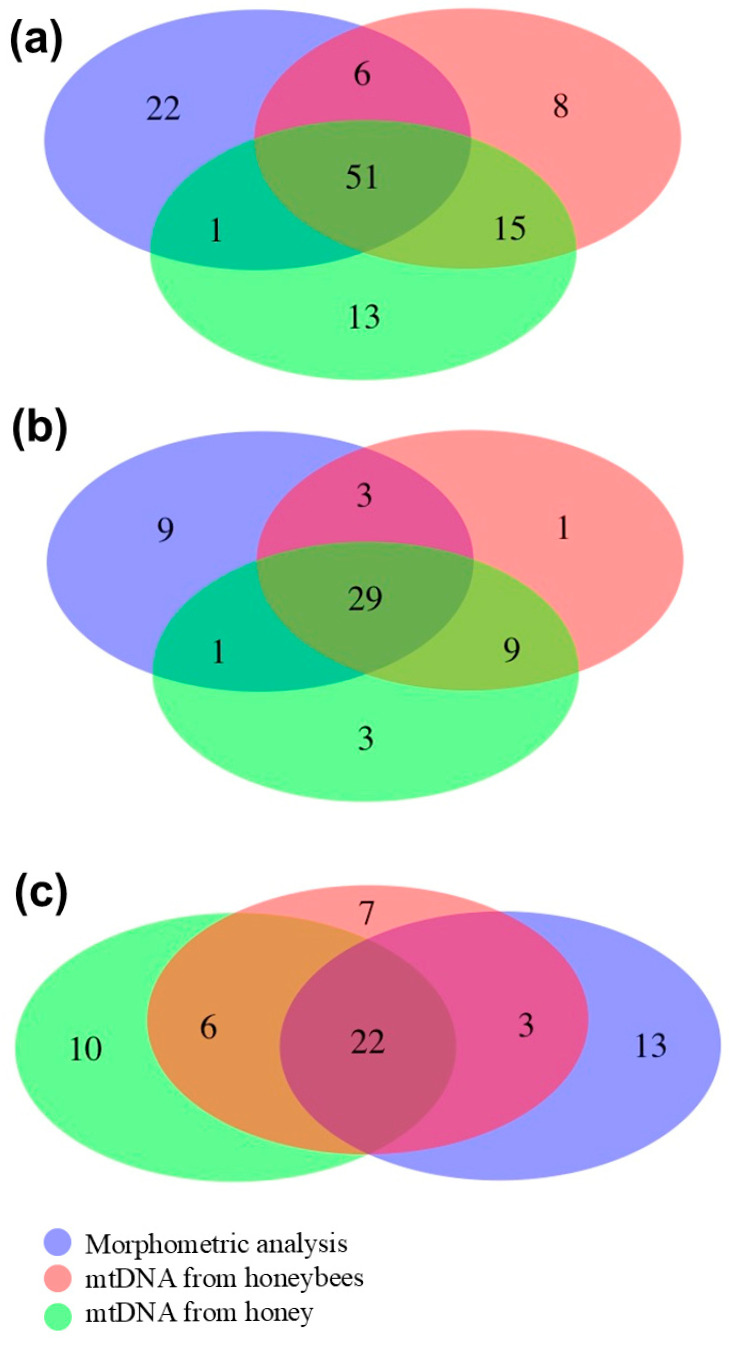
Venn diagrams based on the results obtained using the three methods to detect “compatible” colonies being assigned to *A. m. ligustica* in (**a**) Dataset 1, (**b**) Sub-dataset 2, and (**c**) Sub-dataset 3. The three methods used for the assignment of the colonies are reported with different colors.

Figure 3 displays Venn diagrams based on the results obtained using the three methods to detect compatible colonies assigned to *A. m. ligustica* with the three datasets. Table S3 presents pairwise matched information divided for “compatible” and “non-compatible” colonies, as determined using the same three approaches. When considering the two DNA methods in Dataset 1, there was the largest number of concordant “non-compatible” colonies (n = 5), which represent 71.4% and 41.7% of the “non-compatible” colonies identified by the tRNALeu-COII mtDNA region analysis of the honeybees and the mtDNA analysis of the honey samples, respectively. The same number of concordant “non-compatible” colonies (n = 5) was found in Sub-dataset 3, which represent 83.3% and 55.6% of the “non-compatible” colonies identified by the same two methods reported for Dataset 1, respectively. There was no colony that could be declared “non-compatible” by all three methods together.

Cohen’s Kappa coefficients, which measure the level of agreement between two raters (in this case, two different methods in a pairwise analysis) that each classify items (the colonies) into mutually exclusive categories (i.e., “compatible” and “non-compatible” with *A. m. ligustica*), are reported in Table 2. There was poor agreement (*κ* < 0) between the morphometric analysis and both DNA-based analyses considering all colonies and the two sub-datasets. However, the two DNA-based methods showed substantial agreement (*κ* > 0.61) in the classification of “compatible” and “non-compatible” colonies across all three datasets (Dataset 1, Sub-dataset 2, and Sub-dataset 3).

Bayesian analyses indicated that, in general, there was a lower probability for colonies morphometrically “compatible” with *A. m. ligustica* to have “compatible” mtDNA haplotypes (C1 and/or M3/M7) in their honeybees or honey samples (C1 and M) compared to the opposite scenario (Table 2). This suggests that “compatible” mtDNA information is a better predictor of “compatible” morphometric information than the reverse. The highest probability was observed in Dataset 1 for colonies with “compatible” mtDNA haplotypes in their honeybees, potentially also having “compatible” mtDNA sequences in their honey samples (0.971), indicating a strong consistency between the two mtDNA approaches.

## 4. Discussion

In order to effectively implement conservation programs for honeybee subspecies, it is crucial to be able to monitor the spread of *A. mellifera* genetic resources. The recent approval of a law in the Emilia-Romagna region (Northern Italy) that prohibits the breeding and introduction of subspecies other than *A. m. ligustica* [28] has raised interest in cost-effective analytical approaches capable of discriminating this subspecies from others or hybrid populations. Similar questions and problems have been faced by several authors who monitored the genetic integrity of other honeybee subspecies in various European countries [8,9,10,11,12].

Multiple sources of information as well as their combination can be utilized for discriminating honeybee subspecies. Specific genetic characteristics detected through morphometric and DNA analyses, including mtDNA haplotypes, can be used to describe a honeybee subspecies [1,2,3,8,9,36]. Morphometric analyses can only be conducted after honeybees have been collected and properly prepared to measure several morphometric features of the worker bees. This is a quite demanding method in terms of sample preparation even though the subsequent measurement and statistical steps can be automatized, including the detection of geometric morphometrics of honeybee wings, which can be used for the honeybee subspecies classification [41,42,43]. DNA analysis can be performed directly on the collected honeybees [2,3,36] or on their products, allowing for the detection of honeybee DNA fingerprinting [29,30,31]. In the latter case, the analyzed honey may not only come directly from the honeycombs with a specific origin from a colony (as in this study), but it may also originate from an apiary or from a beekeeper with multiple apiaries, indirectly representing many colonies [29,30,31].

In this study, we compared the results of three different methods used to assign a colony to *A. m. ligustica* subspecies or exclude the assignment to this subspecies. To simplify the interpretation of the results, we referred to the classifications into two categories: “compatible” with *A. m. ligustica* and “non-compatible” with *A. m. ligustica* or “hybrid” between *A. m. ligustica* and other subspecies. The first method involved standard morphometric analysis of worker bees, which categorized colonies as either *A. m. ligustica* or hybrids. The second method analyzed the tRNALeu-COII mtDNA region in honeybees from the same colonies, identifying the presence of the C1 and M3/M7 haplotypes as “compatible” with the assignment to *A. m. ligustica* [3,24]. Therefore, the presence of other mtDNA haplotypes was incompatible with the assignment to this subspecies [3,24,40]. Using honeybees for this analysis could allow for the same sampled bees to be used for morphometric analyses as well, making it practical and cost-effective. Collecting larvae or pupae, which can directly relate to the queen currently in the hive, is not always feasible year-round, or as part of a routine large-scale monitoring approach. The third method examined a short informative fragment of the tRNALeu-COII mtDNA region in honeycomb honey collected from the same colonies, with colony considered “compatible” with *A. m. ligustica* if honey samples only showed the C1 and/or M mtDNA haplotypes (as the M haplotype in this analysis could be the “compatible” M3/M7 haplotypes that this method was not able to distinguish from other M haplotypes [29,31]). The presence of other mtDNA haplotypes or multiple mtDNA haplotypes on the same sample was not consistent with the assignment to this subspecies [3,24,40]. In the DNA analysis of honeybees, we divided the data into two sub-datasets based on the number of honeybees analyzed in each colony: either one worker bee or multiple worker bees. This allowed us to evaluate if multiple worker bees were necessary to obtain a comprehensive picture of the mtDNA haplotypes present in a colony or if sampling only one worker bee randomly would be sufficient for this analysis. To our knowledge, this is the first study that evaluated the possibility to combine information from these three levels for this purpose.

The standard morphometric analysis revealed an initial estimate of the hybridization level in Emilia-Romagna honeybee populations. Approximately 21% of the colonies that we randomly sampled were considered as “hybrids” or “non-compatible” with *A. m. ligustica*. To our knowledge, this is the first published report on the frequency of hybrid populations determined using morphometric information in Northen Italy since Nazzi’s pioneering study over 30 years ago [22]. Nazzi [22] investigated honeybees in Northeastern Italy, an area known for natural hybridization between *A. m. ligustica* and *A. m. carnica*. It is evident that the hybridization detected in the Emilia-Romagna honeybee population is derived from the deliberate introduction of subspecies other than *A. m. ligustica* or hybrid queens due to beekeeping practices, as previously proposed from mtDNA information [30,36]. Emilia-Romagna is geographically distant from the areas where other subspecies are considered native and from zones of natural hybridization (Figure 1). Other studies have utilized morphometric analyses to assess the genetic integrity of honeybee populations, mainly sampled in the *A. m. iberiensis*, *A. m. mellifera*, and *A. m. carnica* original geographic ranges, showing various rates of gene flow from other subspecies or lines, in particular from *A. m. ligustica* and Buckfast hybrids, raising some concerns on the increasing percentage of colonies not classified according to what would be expected (e.g., [44]).

When analyzing the tRNALeu-COII mtDNA region in the 413 honeybees collected from the 80 colonies considered in the current study, we found that 87.9% of the mtDNA haplotypes were “compatible” with *A. m. ligustica* (C1 = 353 sequences; M3/M7 = 10 sequences, even though all from one colony). The frequency of M3/M7 haplotypes was confirmed to be very marginal, as we already reported [36]. The total percentage of “compatible” mtDNA haplotypes was nearly identical to what we reported in a previous study, where we sampled honeybees from over 1100 colonies across the entire Emilia-Romagna region during the same time period [36]. In that study, conducted on different samples than those analyzed here and where one honeybee was analyzed for each colony, the percentage of C1 + M3/M7 mtDNA haplotypes was 87.3% [36]. This value is also similar to the percentage of “compatible” colonies being assigned to *A. m. ligustica* with the same method that we obtained in the current study (~91%). These nearly identical results indicate that the current study, although based on a lower number of sampled colonies, provides a good representation of the genetic characteristics of the honeybee population in the Emilia-Romagna region. Furthermore, this indirectly supports that the sampling of the colonies included in the current study was not biased, despite the lower number investigated. It was also interesting to note that when analyzing multiple honeybees from each colony, the probability of identifying at least one honeybee carrying an alien mtDNA haplotype increased (24%). This excluded the colony from being “compatible” with *A. m. ligustica*, as we defined in this study. This could be due to the increased likelihood of analyzing a honeybee that, for some reason, did not originate from the colony’s queen (perhaps due to drift from nearby colonies in the same apiary) or that resulted from comb exchanges or artificial nucleus formations or replacements of the queens. It is clear that these events or beekeeping practices cannot be known in advance and should be considered when interpreting the results derived from the analysis of only one honeybee per colony. It is worth mentioning that almost all previous studies that monitored the diffusion of honeybee mtDNA haplotypes in various regions in Europe and outside of Europe relied on only one honeybee analyzed per colony (e.g., [11,44,45,46,47,48,49]). Only a few studies analyzed more than one honeybee per colony (two or three), typically to confirm the results (e.g., [9,50]). Ostroverkhova et al. [51] analyzed multiple honeybees to describe the level of mixed mtDNA haplotypes per colony in a Russian hybrid population. These authors showed that approximately 8% of colonies had two or three mtDNA haplotypes, which is nearly the same frequency that we identified in our study when considering only the 38 colonies for which multiple worker bees were analyzed (7.9%). The presence of mixed colonies should be regarded with caution when interpreting the results derived from only one honeybee. Additionally, it should also be considered what could be the minimum number of honeybees that may be analyzed to identify “hybrid” colonies and what could be the minimum proportion of various mtDNA haplotypes to infer the reason for their mixed presence and exclude the possible effect of drift among colonies of the same apiary. It would also be interesting to compare the results obtained from adult honeybees, pupae, and larvae from the same colonies. Pupae and larvae may better reflect the mtDNA line of the queen present in the colony during the sampling. Other studies are necessary to answer these questions in more detail.

The analysis of the honeybee mtDNA traces left in the honey samples supported the results obtained from the direct analysis of mtDNA haplotypes from honeybees, with a slightly lower frequency of “compatible” colonies (85%). This lower frequency may be due to the more complex or comprehensive origin of the honey samples, even if they were collected from honeycombs sampled from the same colonies. Environmental DNA present in the honey may better provide a snapshot of the genetic composition of the colony, as many more honeybees that those analyzed individually contributed to the production of honey. Results from previous studies [30,31] on about 100 honey samples produced in the Emilia-Romagna region during approximately the same period (2018–2020) confirmed that the C1 haplotype was the most frequent. Nearly all samples contained this mtDNA haplotype, which was also the only haplotype identified in 66% of honey produced in this region in 2018 [30]. It is worth highlighting that in these other studies [30,31], honey samples were not linked to a single colony, as in the current study. Therefore, they may have captured traces from multiple colonies, increasing the likelihood of being enriched with traces from “non-compatible” mtDNA haplotypes.

Agreements were reached among all three methods in assigning a colony to *A. m. ligustica* for the majority of colonies (~64%), indicating that their multi-level features may provide, at least in part, consistent information in field trials. However, in approximately more than one-third of colonies, agreements were not reached using all three methods. This is likely due to intrinsic differences in the diagnostic informativeness of morphometric and DNA-based analyses targeting different matrices (i.e., honeybees and honey), which may be less or more sensitive in capturing the genetic history and background of the colonies. The level of agreement in classifying the colonies between morphometric analysis and the DNA methods was poor, as defined by the Cohen’s Kappa statistic. This further indicates intrinsic differences provided by each method. A higher level of agreement was observed between the two DNA methods (82.5%, with high Cohen’s Kappa coefficients), due to their shared reliance on mtDNA information, despite being detected at different levels. Other studies have reported inconsistent assignment of colonies to specific subspecies when morphometric analysis has been supplemented with information on honeybee mtDNA lineages, suggesting the influence of past introgression events in determining within-population mtDNA haplotype heterogeneity [11,12,44,48]. It was also interesting to note that a morphometrically compatible colony had a lower probability to have compatible mtDNA haplotypes than the opposite scenarios. This may indicate that, at least in part, the Emilia-Romagna honeybee population has experienced introgression of maternal lineages mainly carrying C2 and A mtDNA haplotypes not derived from *A. m. ligustica* in a genetic background primarily constituted by *A. m. ligustica* lineages. This would be incompatible with a generally accepted definition of *A. m. ligustica* subspecies colonies that morphometrically may be assigned to this subspecies but at the mtDNA level they derive from queens with mtDNA different from that commonly accepted to be characteristic of this subspecies [24]. It is, therefore, important to maintain monitoring of the diffusion of alien mtDNA haplotypes in this region, as we previously suggested due to the relatively high frequency of mtDNA lineages not considered to represent a genetic feature of *A. m. ligustica* [36]. However, in this context, to support sustainable conservation of the genetic integrity of honeybee subspecies, it is also important to consider the combined effect of conservation strategies and beekeeping practices, which have specific breeding objectives that can substantially modify honeybee population structures [7,52,53]. Integrated breeding and conservation strategies need to balance the maintenance of a certain level of within-population genetic diversity with the retention of adaptive potential while also selecting for desirable traits [7,52,53].

It would also be interesting to complete the genetic characterization of the investigated colonies with information at the nuclear genome level to evaluate the extent of introgression at this level compared to other levels. Other studies using whole-genome sequencing data or high-throughput genotyping of single-nucleotide polymorphisms can complement the picture we obtained in this study [19,54,55]. Nuclear genomic data can be produced by analyzing directly single collected honeybees [18], multiple honeybees in DNA-pool sequencing [54], or using honey as a source of honeybee DNA, as we already demonstrated [19]. However, conducting these additional analyses could increase costs and make monitoring programs of honeybee genetic resources on a large scale too expensive.

An important issue that may complicate the interpretation of the results derived from analyzing matrices collected at the colony level is due to several beekeeping practices that tend to lose the direct link between the queen genetics and the genetic features that can be detected by collecting worker bees or honey from honeycombs. This may be one of the reasons for non-concordant results among the three different levels we investigated, which could be in the order of approximately 8%, as indicated by the presence of mixed colonies in our study and in other studies [51], but that could be modified according to the general beekeepers’ practices. Direct analysis of material from the queens could be possible [56] even though it is not very simple and practical to be implemented at large scales. However, it should be taken into consideration that routine monitoring assays should be cost-effective and practical in terms of implementation at a large scale if useful for developing conservation programs of autochthonous honeybee genetic resources. Thus, honey could also be considered the matrix of choice in this context, particularly if applicable at the apiary level (or at the beekeeper level analyzing honey sampled during the production season) to have a complete picture of the mtDNA haplotypes present in an apiary (or in multiple apiaries owned by the same beekeeper) and evaluate if more specific actions would be needed at the colony level based on more precise and direct methods.

## 5. Conclusions

The results obtained in this study further supported the indication that the Emilia-Romagna honeybee population experienced introgression of maternal lineages carrying mtDNA haplotypes not derived from *A. m. ligustica* in a genetic background primarily constituted by this subspecies. Investigating a larger number of colonies could also further support our findings. Additionally, this study provided field data indicating only partial concordance in assigning a colony to *A. m. ligustica* derived from the three applied methods: morphometric analysis and two mtDNA-based methods. It appears that, based on the definition of *A. m. ligustica*, using only one method would increase the error in declaring a colony “compatible” when it is not. The use of mtDNA information alone may have a higher probability of identifying morphometrically “compatible” colonies than the opposite. Therefore, to implement a large-scale and cost-effective approach to determining the honeybee subspecies at the colony level, monitoring mtDNA haplotypes in the honeybee population (either using honeybees or honey as sources of honeybee DNA) could be considered. Although not completely effective, this could serve as an initial and practical compromise to begin initiatives aimed at preserving the genetic integrity of *A. m. ligustica* in the Emilia-Romagna region, as well as in other regions where it is necessary to preserve the genetic integrity of autochthonous honeybee populations. In particular, mtDNA analysis on honey samples may provide a convenient approach, considering the substantial agreement of the results of this method with those from honeybee mtDNA analysis.

## Figures and Tables

**Table 1:** Number of “compatible” and “non-compatible” colonies to the *A. m. ligustica* subspecies (and frequencies), as determined from the morphometric and from the two DNA-based methods.

Methods ^1^	Dataset 1 ^2^		Sub-Dataset 2 ^2^		Sub-Dataset 3 ^2^	
	Comp. ^3^	Non-Comp. ^3^	Comp.	Non-Comp.	Comp.	Non-Comp.
**Morphometric analysis**	63 (0.79)	17 (0.21)	33 (0.79)	9 (0.21)	30 (0.79)	8 (0.21)
**mtDNA from honeybees**	73 (0.91) ^ab^	7 (0.09)	41 (0.98) ^a^	1 (0.02)	32 (0.84) ^b^	6 (0.16)
**mtDNA from honey**	68 (0.85)	12 (0.15)	39 (0.93)	3 (0.07)	29 (0.76)	9 (0.24)

^1^ Methods used for the assignment of the colonies. ^2^ Considered dataset and sub-datasets. ^2^ Dataset 1 included all colonies (n = 80), Sub-dataset 2 included 42 colonies, and Sub-dataset 3 included 38 colonies. ^3^ “Compatible” (comp.) or “non-compatible” (non-comp.) with the assignment to *A. m. ligustica* based on morphometric information or based on the DNA information from honeybees or honey. Different superscripts (a,b) indicate a significant difference (*p* < 0.05) in a two-tailed Fisher exact test between pairs of datasets within the method.

**Table 2:** Statistical differences and Cohen’s Kappa statistic for the three datasets between pairs of methods in terms of the identification of colonies that are “compatible” and “non-compatible” with *A. m. ligustica*, as well as Bayesian posterior probabilities in assigning a colony to *A. m. ligustica* based on information from one method when referred to a second method.

Datasets	Method ^1^	Fisher Exact Test/Cohen’s Kappa ^2^			Bayesian Analyses ^3^		
		Morph.	mtDNA Honeybees	mtDNA Honey	Morph.	mtDNA Honeybees	mtDNA Honey
**Dataset 1**	**Morphometric**	-	−0.046 (0.086)	−0.130 (0.081)	-	0.905	0.825
	**mtDNA from honeybees**	0.053	-	0.659 (0.128)	0.781	-	0.904
	**mtDNA from honey**	0.412	0.329	-	0.765	0.971	-
**Sub-dataset 2**	**Morphometric**	-	−0.045 (0.042)	−0.120 (0.056)	-	0.970	0.909
	**mtDNA from honeybees**	0.015	-	0.641 (0.235)	0.780	-	0.927
	**mtDNA from honey**	0.010	0.116	-	0.769	0.882	-
**Sub-Dataset 3**	**Morphometric**	-	−0.046 (0.149)	−0.015 (0.159)	-	0.833	0.733
	**mtDNA from honeybees**	0.768	-	0.689 (0.140)	0.781	-	0.875
	**mtDNA from honey**	1.000	0.566	-	0.759	0.793	-

^1^ Methods used for the assignment of the colonies. ^2^ Below the diagonal: *p*-value of the two tailed Fisher exact test between pairs of methods (identified with the intersection between the rows and columns) counting the number of “compatible” and “non-compatible” colonies that are determined independently. Above the diagonal: Cohen’s Kappa coefficient (and in parenthesis the standard error) between pairs of methods (identified with the intersection between the rows and columns) counting the number of “compatible” and “non-compatible” colonies that are determined independently. ^3^ Posterior probability defined between pairs of methods: below the diagonal, the posterior probability of the method in the column against the method in the rows; above the diagonal, the posterior probability in the reverse direction.

## Data Availability

Data are reported in the text and in the Supplementary Materials. Detailed information on the results for each colony obtained from the three methods is available in the following repository: https://doi.org/10.5281/zenodo.14725580.

## Supplementary Materials

The following supporting information can be downloaded at: https://www.mdpi.com/article/10.3390/ani15121743/s1. Table S1: Detailed information on the analyzed honeybee colonies. Table S2: Morphometric values (mean and standard deviation) used for the identification of the *A. m. ligustica* subspecies (Source: Regulation of the National Register of Italian Honeybee Breeders—Disciplinare Dell’Albo Nazionale Degli Allevatori di Api Italiane [34]). Table S3: Pairwise matched information for compatible (below the diagonal) and non-compatible (above the diagonal) colonies as being assigned to *A. m. ligustica*, as determined using the three approaches. The diagonal reports the total number of compatible and non-compatible colonies (written in italics) determined using the three methods. Figure S1: The right forewing of a honeybee, indicating the 19 landmark points (numbered from 0 to 18) acquired and used for morphometric analyses. These points were used to calculate the following 30 parameters: Angle A1, Angle A4, Angle B3, Angle B4, Angle D7, Angle E9, Angle G7, Angle G18, Angle H12, Angle J10, Angle J16, Angle K19, Angle L13, Angle M17, Angle N23, Angle O26, Angle Q21, Radial Field, Length A, Length B, Length C, Length D, Inner Length, Inner Width, Discsh, Index Cubital, Index Precubital, Index Dumbbell, Index Radial, and Area 6.

## Abbreviations

The following abbreviations are used in this manuscript:

mtDNA	Mitochondrial DNA
PCR	Polymerase chain reaction
