# Supplementary material for: Comparing Morphometric and Mitochondrial DNA Data from Honeybees and Honey Samples for Identifying Apis mellifera ligustica Subspecies at the Colony Level
Source: Animals (Basel). 2025 Jun 12;15(12):1743. doi: 10.3390/ani15121743 (PMC12189643; doi:10.3390/ani15121743)
Supplement: Supplementary file 1 [file animals-15-01743-s001.zip › animals-3635555-supplementary.pdf]

animals

MDPI

Article

Supplementary Material

Comparing Morphometric and Mitochondrial DNA Data from Honey Bees and Honey Samples for Identifying *Apis mellifera ligustica* Subspecies at the Colony Level

Valeria Taurisano, Anisa Ribani, Valerio Joe Utzeri, Dalal Sami, Kate Elise Nelson Johnson, Giovanni Formato, Marcella Milito, Giuseppina Schiavo, Samuele Bovo, Francesca Bertolini, Luca Fontanesi

Table S1. Detailed information on the analyzed honey bee colonies.

Colony ID	Beekeeper ID	Administrative Province	N. of analysed honey bees
1	1	Bologna	1
2	1	Bologna	1
3	1	Bologna	1
4	2	Ferrara	1
5	2	Ferrara	10
6	2	Ferrara	1
7	3	Bologna	8
8	3	Bologna	1
9	3	Bologna	10
10	4	Bologna	1
11	4	Bologna	8
12	4	Bologna	1
13	5	Modena	1
14	5	Modena	8
15	5	Modena	10
16	6	Bologna	1
17	6	Bologna	10
18	6	Bologna	1
19	7	Piacenza	17
20	7	Piacenza	10
21	7	Piacenza	1
22	8	Piacenza	9

23	8	Piacenza	10
24	8	Piacenza	10
25	9	Piacenza	8
26	9	Piacenza	10
27	9	Piacenza	10
28	10	Rimini	10
29	10	Rimini	1
30	10	Rimini	10
31	11	Rimini	1
32	11	Rimini	1
33	11	Rimini	10
34	12	Bologna	1
35	12	Bologna	10
36	12	Bologna	1
37	13	Bologna	1
38	13	Bologna	1
39	13	Bologna	1
40	13	Bologna	7
41	13	Bologna	1
42	13	Bologna	1
43	14	Reggio Emilia	9
44	14	Reggio Emilia	10
45	14	Reggio Emilia	1
46	15	Parma	10
47	15	Reggio Emilia	1
48	15	Reggio Emilia	10
49	16	Ravenna	10
50	17	Ravenna	10
51	17	Ravenna	1
52	17	Ravenna	1
53	18	Bologna	10
54	18	Bologna	1
55	18	Bologna	1
56	19	Ferrara	10
57	19	Ferrara	1
58	19	Ferrara	1
59	20	Ferrara	1
60	20	Ferrara	10
61	20	Ferrara	10
62	21	Ferrara	1
63	21	Ferrara	1
64	21	Ferrara	9
65	22	Piacenza	8

66	22	Piacenza	10
67	22	Piacenza	1
68	23	Parma	1
69	23	Parma	10
70	23	Parma	1
71	24	Modena	10
72	24	Modena	1
73	24	Modena	1
74	25	Reggio Emilia	10
75	26	Parma	10
76	26	Parma	1
77	26	Parma	1
78	27	Parma	1
79	27	Parma	10
80	27	Parma	1

Table S2. Morphometric values (mean and standard deviation) used for the identification of the *A. m. ligustica* subspecies (Source: Regulation of the National Register of Italian Honey Bee Breeders - Disciplinare Dell'Albo Nazionale Degli Allevatori di Api Italiane [34]).

<table>
<tr><th colspan="2">Morphometric traits</th><th>Unit</th><th>Mean</th><th>Standard Deviation</th></tr>
<tr><td colspan="2">Pigmentation of the third tergite</td><td>class</td><td>7.65</td><td>0.73</td></tr>
<tr><td rowspan="16">Right forewing</td><td>Length</td><td>mm</td><td>9.17</td><td>0.12</td></tr>
<tr><td>Width</td><td>mm</td><td>3.23</td><td>0.06</td></tr>
<tr><td>Cubital venation a</td><td>mm</td><td>0.56</td><td>0.03</td></tr>
<tr><td>Cubital venation b</td><td>mm</td><td>0.24</td><td>0.02</td></tr>
<tr><td>Cubital Index</td><td>-</td><td>2.44</td><td>0.24</td></tr>
<tr><td>Angle A4</td><td>degrees</td><td>30.55</td><td>1.18</td></tr>
<tr><td>Angle B4</td><td>degrees</td><td>109.03</td><td>2.73</td></tr>
<tr><td>Angle D7</td><td>degrees</td><td>98.78</td><td>2.12</td></tr>
<tr><td>Angle E9</td><td>degrees</td><td>23.52</td><td>1.19</td></tr>
<tr><td>Angle G18</td><td>degrees</td><td>91.17</td><td>2.42</td></tr>
<tr><td>Angle J10</td><td>degrees</td><td>52.26</td><td>1.91</td></tr>
<tr><td>Angle J16</td><td>degrees</td><td>95.52</td><td>2.39</td></tr>
<tr><td>Angle K19</td><td>degrees</td><td>78.68</td><td>1.90</td></tr>
<tr><td>Angolo L13</td><td>degrees</td><td>13.62</td><td>1.05</td></tr>
<tr><td>Angle N23</td><td>degrees</td><td>92.74</td><td>1.96</td></tr>
<tr><td>Angle 026</td><td>degrees</td><td>35.73</td><td>1.97</td></tr>
</table>

Table S3. Pairwise matched information for compatible (below the diagonal) and non-compatible (above the diagonal) colonies as being assigned to *A. m. ligustica* as determined using the three approaches. The diagonal reports the total number of compatible and noncompatible colonies (written in italics) determined using the three methods.

**Datasets**	**Methods**[1]	**Morphometric**	**mtDNA from honey bees**	**mtDNA from honey**
**Dataset 1**	Morphometric	*63/7*	1	1
	mtDNA from honey bees	57	*73/7*	5
	mtDNA from honey	52	66	*68/12*
**Sub-dataset 2**	Morphometric	*33/9*	0	0
	mtDNA from honey bees	32	*41/1*	0
	mtDNA from honey	30	38	*39/3*
**Sub-Dataset 3**	Morphometric	*30/8*	1	1
	mtDNA from honey bees	25	*32/6*	5
	mtDNA from honey	22	28	*29/9*

[1]Methods used for the assignment of the colonies: Morphometric analysis, analysis of the tRNALeu-COII mtDNA region from honey bees (mtDNA honey bees), analysis of the honey bee mtDNA region that can distinguish from A, C1, C2 and M mtDNA lineages from honey DNA (mtDNA honey).

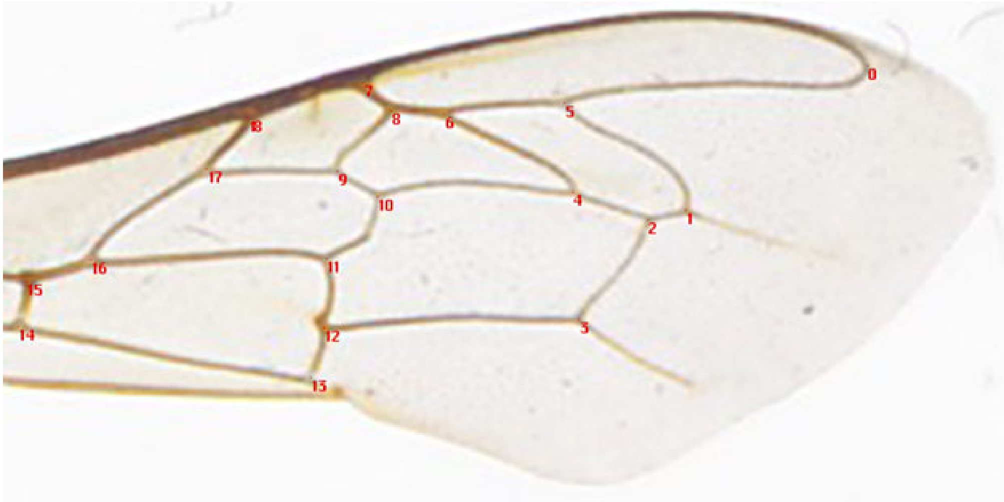

Figure S1. The right forewing of a honey bee with indicated the 19 landmark points (numbered from 0 to 18) acquired and used for morphometric analyses. These points were used to calculate the following 30 parameters: Angle A1, Angle A4, Angle B3, Angle B4, Angle D7, Angle E9, Angle G7, Angle G18, Angle H12, Angle J10, Angle J16, Angle K19, Angle L13, Angle M17, Angle N23, Angle O26, Angle Q21, Radial Field, Length A, Length B, Length C, Length D, Inner Length, Inner Width, Discsh, Index Cubital, Index Precubital, Index Dumbbell, Index Radial, Area 6.
